# Wideband Epidermal Antenna for Medical Radiometry

**DOI:** 10.3390/s20071987

**Published:** 2020-04-02

**Authors:** Germán León, Luis F. Herrán, Ignacio Mateos, Enrique Villa, Juan B. Ruiz-Alzola

**Affiliations:** 1 Department of Electrical Engineering, Group of Signal Theory and Communications, Universidad de Oviedo, 33202 Gijón, Spain; herranluis@uniovi.es; 2 Applied Magnetism and Optics Group, Universidad de Cádiz, 11519 Cádiz, Spain; ignacio.mateos@uca.es; 3 IACTec Medical Technology Group, Instituto de Astrofísica de Canarias (IAC), 38205 San Cristóbal de La Laguna, Spain; enrique.villa.benito@iac.es (E.V.); juan.ruiz@ulpgc.es (J.B.R.-A.); 4 Instituto Universitario de Investigación Biomédica y Sanitaria (IUIBS), Universidad de Las Palmas de Gran Canaria (ULPGC), 35001 Las Palmas de Gran Canaria, Spain

**Keywords:** epidermal antenna, flexible wideband antenna, microwave medical applications

## Abstract

Microwave thermometry is a noninvasive and passive technique for measuring internal body temperature. Wearable compact antennas, matched to the specific body area, are required for this method. We present a new epidermal wideband antenna for medical radiometry. The double asymmetric H-shaped slot antenna was designed to be matched to different parts of the body without fat layers. The slots are fed by a short-circuited microstrip line in order to decrease size and back radiation, thus reducing potential interferences. In this way, contribution to radiometric temperature due to back radiation is lower than 4%, versus the 20% of the volume under investigation, over the whole operating frequency band. The designed prototype was manufactured on a flexible substrate. The antenna is a very small size, to make it comfortable and suitable for being used by patients with different body mass indexes. The double H-shaped antenna shows good wideband matching results from around 1.5 GHz up to 5 GHz, in different body locations such as the neck, foot instep and foot sole.

## 1. Introduction

**Figure 1:**
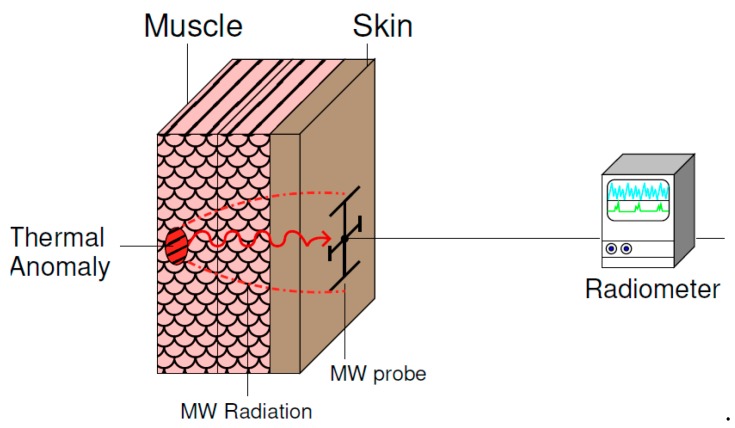
Scheme of the proposed MMR measurement set-up.

Medical Microwave Radiometry (MMR) is a low-cost, non-ionizing, non-invasive procedure for medical diagnosis. This passive method is based on the measurement of the internal thermal electromagnetic (EM) radiation of a body region, and its detection using a radiometer system (Figure 1). Hence, the analysis of any anomalous temperature variation measured using these systems could help in the assessment of a medical complication or pathology. Indeed, MMR has already been applied to cancer detection [1], cancer treatment [2], hyperthermia temperature control [3], human core temperature tracking [4], atherosclerosis in the human carotid [5] and the diagnosis of arterial disease in diabetic patients [6]. In all these applications, the MMR system has to be able to detect temperature variations of less than 1 K, and thus, very small changes in the received power. Therefore, an antenna able to measure the EM radiation in an efficient way is required. Hence, it demands a good coupling between the MMR probe and the area under test. In addition, a good matching is also compulsory, which depends on the specific body region and on the patient’s complexion, since an adipose layer close to the skin could significantly modify the permittivity of the intended tissue. This is mainly due to the substantially lower value of the relative permittivity of fat tissue compared to skin or muscle.

A great variety of antennas have been developed for medical diagnosis, depending on the body region under test and the working band. Folded antennas are used to reduce size in off-body wideband systems [7,8,9]. However, the distance between the antenna and the body is critical for good matching, and they show a poor front-to-back ratio. To overcome these problems and improve the coupling, on-body matched antennas are chosen not only for medical diagnosis [10,11,12,13] but also for Wireless Personal Area Network (WPAN) communications [14,15]. In order to increase the efficiency of the measurement system, most of these designs have a reflector to reduce the backward radiation [10,14], but this also increases the complexity. Moreover, wideband epidermal antennas can help MMR, since they can be used to measure below skin surface in multiband applications [3]. Besides, they can help to increase the matching between the device and the subject under test to a larger number of patients [5,6]. However, these antennas are not suitable for wearable devices. 

In this work, an epidermal antenna, based on an asymmetric double-H antenna, is proposed for MMR applications, with the goal of evaluating diabetic foot [6] or carotid artery diseases associated with subcutaneous temperature anomalies [5] specifically. The radiating slots are matched through a short-circuited stub to body regions without adipose layers, and the design does not require reflector planes or intermediate matching media to reduce back radiation. Low-profile and easy to manufacture designs have been achieved, so this antenna is a potential candidate as a wearable antenna. The design and radiation characteristics are described in Section 2. A measurement campaign was carried out (Section 3) to show the matching of the antenna in several scenarios between 1.5 and 5 GHz, as well as the low back radiation level. Finally, the main conclusions are summarized in Section 4.

## 2. Wideband Antenna Design

### 2.1. Short-Circuited Stub Matching

**Figure 2:**
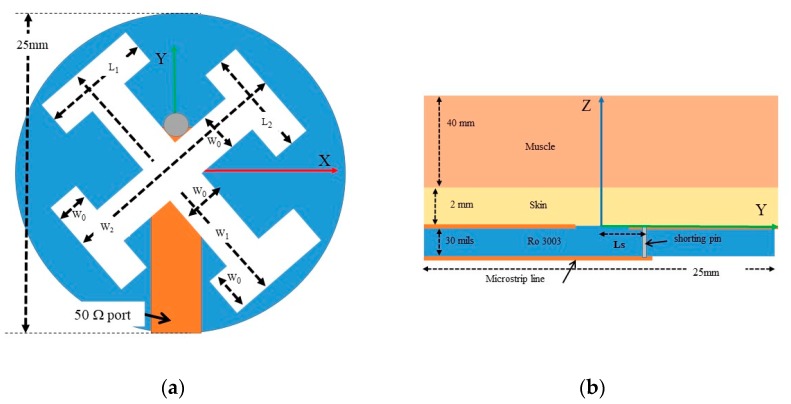
Scheme of the asymmetric Jerusalem-cross slot antenna. (**a**) Top view, (**b**) side view.

A rectangular slot, placed on the body, can resonate if its length is approximately

(1) $$W_{s}=\frac{\lambda_{body}}{2}=\frac{c_{0}}{2f\sqrt{\epsilon_{body}}}$$

where $\lambda_{body}$ and $\epsilon_{body}$ are the effective wavelength and relative permittivity of the body, respectively, and *f* the operating frequency. In that case, the slot mainly radiates towards the body due to the high contrast between the permittivities of the air and the body. In order to decrease the size of the radiating antenna, an H-shaped slot can be used [16]. Subsequently, a Jerusalem-cross slot can be formed by the combination of two H-shaped slots. A schematic diagram of the proposed antenna is illustrated in Figure 2. The slots are fed by a microstrip line ending in a short circuit to reduce the back radiation, instead of a typical open circuit that produces spurious radiation. The feed line, in the front side of the substrate, and the slots in the backside, form an angle of 45 degrees, to excite both possible resonances. The antenna has been manufactured inside a 25-mm diameter circle, which is small enough to be comfortable for patients. Variations on the ground plane size do not generate changes in the antenna behavior.

The antenna must be matched to a human neck [5] or foot [6], which are body areas without an adipose layer. Therefore, a two-layer structure was chosen to model these body volumes. The antenna was in contact with a 2-mm thick skin layer. The second layer (muscle) was modeled as a large enough volume to be considered semi-infinite (Figure 2b). Then, a 40-mm thickness was considered. Since the dielectric properties of the tissues change with frequency, according Table 2 [17], several simulations were carried out using these parameters. The antenna was designed on a Rogers3003^TM^ flexible substrate, with relative permittivity $\epsilon_{r}=3.0$, loss tangent tanδ=0.0013 at 10 GHz, and a thickness of h=30 mils. The design parameters of the antenna were optimized with HFSS [17] and are shown in Table 1. The set of parameters $W_{1}+L_{1}$ and $W_{2}+L_{2}$ control the resonant frequencies of each slot, in agreement with Equation (1), while the parameters $W_{0}$ and $L_{s}$ adjust the matching between the antenna and the body region. In the simulations, a frequency-dependent Debye model was used, with the dielectric properties given in Table 2. 

**Figure 3:**
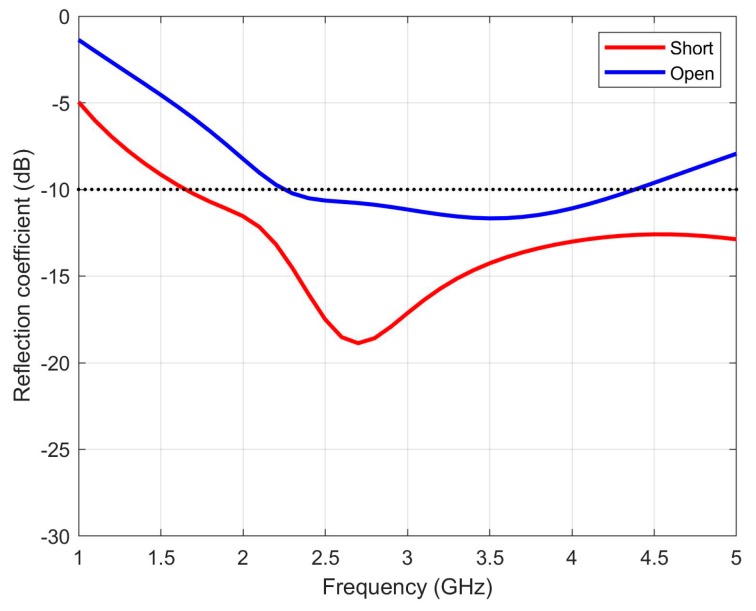
Simulated reflection coefficients of the asymmetric Jerusalem cross slot antenna.

In order to show the better performance of a short-circuited stub versus an open-ended version, the input reflection coefficients of the antenna, using both configurations, are compared in Figure 3. The parameter $L_{s}$ was fitted to the maximum value for the open stub configuration, that is, *L_s_* = 12 mm. The short-circuited antenna shows a good matching from 1.5 GHz up to more than 5 GHz. This result is much better than the open stub design. To improve the matching for the open-circuited antenna, the surface of the antenna has to be enlarged, reducing its potential use as an epidermal antenna.

### 2.2. Losses in the Volume

In [4], a microwave radiometer model for body temperature is sketched. In this approach, the temperature of each layer is weighted by a function $W_{i}$, which depends on the frequency and conductivity of each tissue layer. Then, the whole body temperature defined by this model is expressed as

(2) $$T=\overset{N}{\underset{i=1}{\sum }}W_{i}T_{i}$$

where *T_i_* is the *i*-th layer, *N* is the total number of layers, and *T* is the radiometric temperature at the probe. The weighting functions can be quantified, by reciprocity, as the relation between the power absorbed by the layer ($P_{di}$), integrating the volume power loss density of each layer ($D_{i}$) and the total power (*P*) provided by the feed. The weighting function of a layer placed between 0 < *Z*_1_ < *Z*_2_ can be calculated as

(3) $$W_{i}=\frac{P_{di}}{P}=\frac{\int_{Z_{1}}^{Z_{2}}\int_{-\infty }^{\infty }\int_{-\infty }^{\infty }D_{i}\left(x,y,z\right)dxdydz}{P}$$

It has to be highlighted that in this equation, the matching of the antenna is taken into account. 

In [4], back radiation contribution is not taken into account. However, it is easy to add another term to Equation (2) to estimate the contribution of the temperature radiation of the lab $\left(T_{lab}\right)$, with a weighting function $W_{back}$, integrating the radiation at a semi-sphere in the open air:

(4) $$W_{back}=\frac{P_{back}}{P}=\frac{\int_{0}^{2\pi }\int_{\pi /2}^{\pi }\frac{{\left|E\left(R,\theta ,\phi \right)\right|}^{2}}{2\eta_{0}}R^{2}sin\theta d\theta d\phi }{P}$$

**Figure 4:**
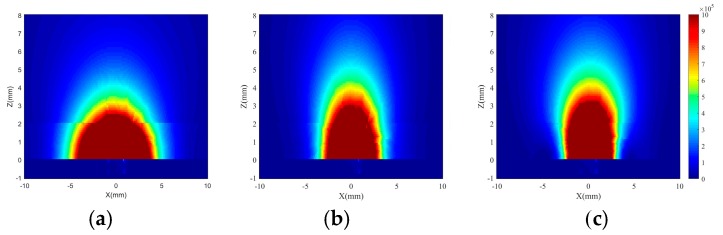
Simulated volume loss density (W/m^3^) in the plane Y = 0 of the asymmetric Jerusalem cross antenna, fed by a power of 1 W at (**a**) 2 GHz, (**b**) 3 GHz, and (**c**) 4 GHz.

In Figure 4, volume power losses for different frequencies inside the proposed phantom model tissues (Figure 2b) are shown in plane Y = 0. In all frequencies, the antenna is fed by a power of 1 W. Although the propagation losses inside the tissue increase with frequency, the E-field remains approximately constant. This is because the electrical size of the antenna also increases with the frequency, and thus the antenna directivity. Similar results were found for the plane *X* = 0. 

These figures also show that the losses inside the substrate (0 < *Z* < −30 mils) are negligible. The electromagnetic properties of the tissues (skin and muscle) are very similar, so a slight discontinuity can be seen at *Z* = 2 mm.

**Figure 5:**
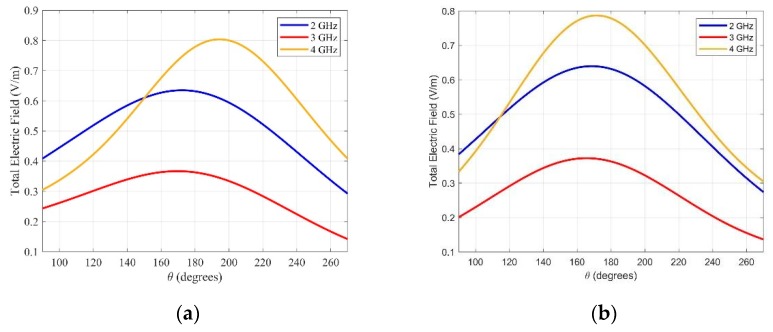
Back radiation electric field of the asymmetric Jerusalem cross antenna for (**a**) ϕ=0° and (**b**) ϕ=90° at different frequencies.

The simulated back radiation total electric field for the two principal cuts of the wideband antenna results are shown in Figure 5. The back radiation of the antenna increases at frequencies higher than 4 GHz due to the bigger slot (corresponding to *W*_1_ and *L*_1_ parameters), and begins to radiate to the free space. The back radiation gain has a minimum of 3 GHz and remains below −18 dBi in the whole band. These results ensure very low interferences coming from outside the tissues, between 1.5 GHz and 4 GHz.

With these values, the weighting functions of each contribution can be calculated, using Equations (3) and (4). We consider our volume under investigation (VUI) to be the muscle slab from depth 3 mm to 7 mm. The whole model [5] consists of a deeper slab (depth > 7 mm), the VUI (depth 3–7 mm) and the skin (depth < 3 mm). We show the simulated weights corresponding to the contribution of each slab, and the back radiation for the temperature formation model (Equation (2)) in Table 3, along with the simulated specific absorption rate (SAR) in the skin, which may be taken as a reference for future applications with radiating antennas. SAR has been calculated for the skin tissue (with density of 1125 Kg/m^3^) when the antenna is fed with a power of 15 mW. This power is within SAR values used in the standard presented in [19] at this frequency band.

As expected, the higher losses are found in the superficial layer. This value increases with frequency. The contribution of the VUI is enough to detect temperature changes of about 1 K. The back radiation contribution remains low enough, so changes in laboratory temperature do not influence the radiometric measurement. From 4 GHz, the back radiation losses increase, and the contribution of the VUI decreases. For this reason, it is recommending to use the probe up to 4 GHz for medical radiometry, although the antenna is matched.

## 3. Experimental Validation

**Figure 6:**
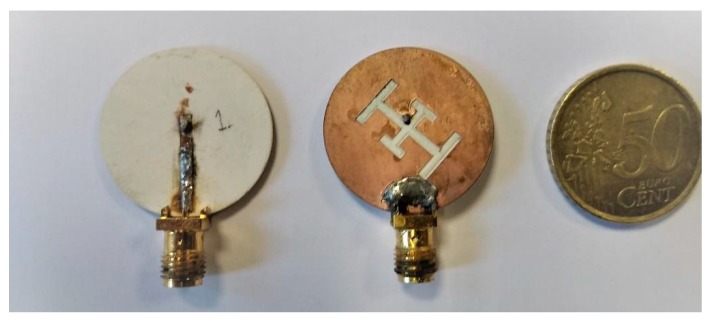
Manufactured prototypes.

Two antennas have been manufactured to validate the characteristics of the described design (Figure 6). In order to demonstrate the matching of the antenna, measurements on four volunteers with different body mass indices (BMI, Table 4) have been performed to show the behavior of the antenna on the body area under test.

### 3.1. Matching Measurement Campaign

**Figure 7:**
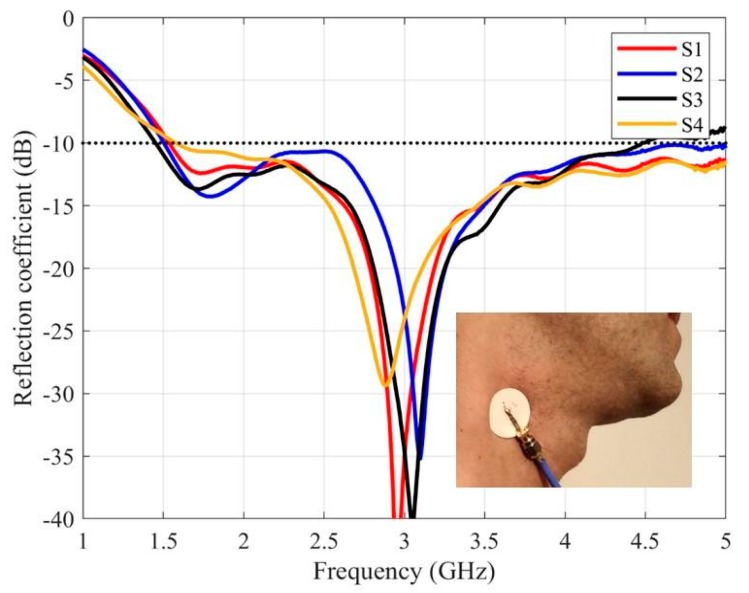
The measured reflection coefficients of the purposed antenna placed on the carotid.

**Figure 8:**
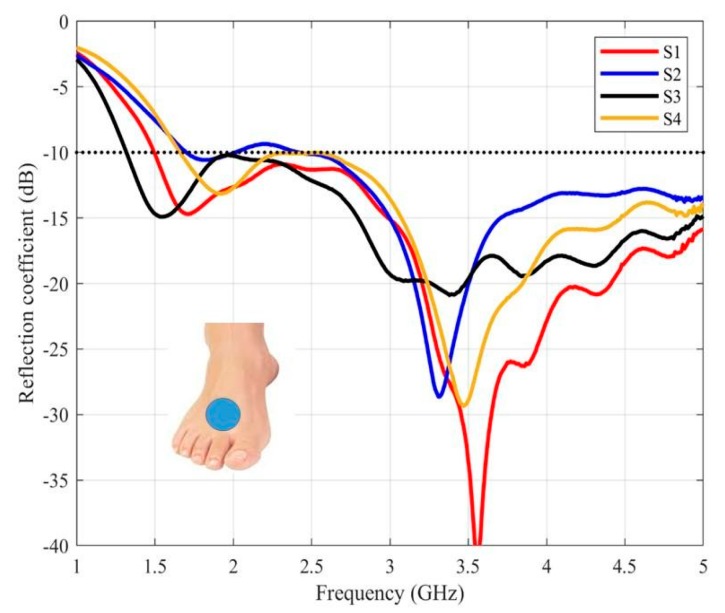
The measured reflection coefficients of the purposed antenna placed on the instep.

**Figure 9:**
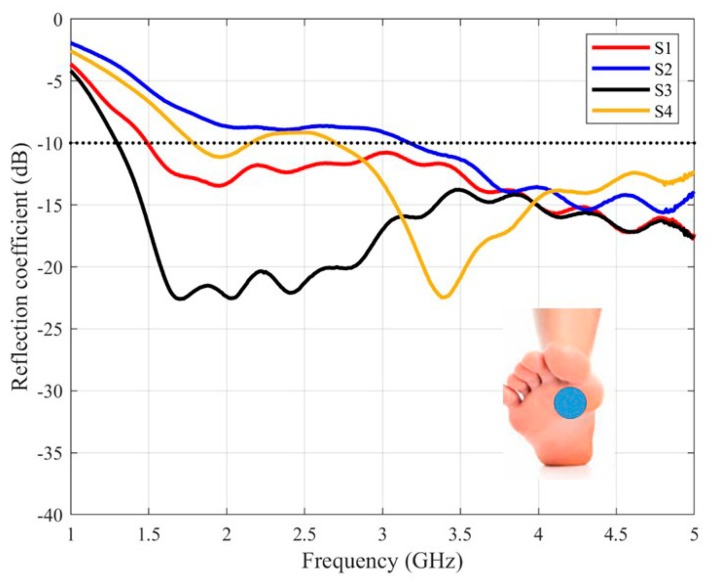
The measured reflection coefficients of the purposed antenna placed on the sole.

The measured reflection coefficients of the antenna are presented in Figure 7, Figure 8 and Figure 9, for each one of the mentioned body areas.

The behavior of the antenna on the carotid (Figure 7) is very similar for the four volunteers. In all cases, the antenna is well matched in the whole required band (between 1.5 GHz and 5.0 GHz), with values below −10 dB between 1.6 GHz to 4.5 GHz in all cases. Moreover, the measured results are very similar to the simulated ones. 

In Figure 8, results for the instep are shown. In this case, the antenna is also matched in three cases, from 1.8 GHz, while for the S2 volunteer, the antenna is slightly mismatched around 2.2 GHz.

The more significant difference has appeared in the sole area (Figure 9) for one of the volunteers (S2). In this particular case, the subject under test presents the smallest foot (Table 4) which hinders the matching of the antenna.

### 3.2. Back radiation Measurement Campaign

**Figure 10:**
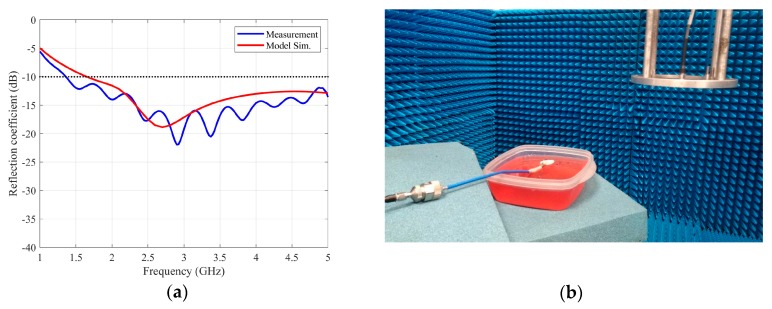
(**a**) Measurement and simulation of the reflection coefficient of the antenna under test (**b**) Back radiation measurement set-up.

In order to show the back radiation level of the designed antenna, an *ex profeso* measurement set-up has been developed. The antenna under test was placed on a mimic gel made with MSL900V2 solution [20] with ε_r_ = 46.50 and σ = 2.28 S/m at 2 GHz, agar-agar as a gelling agent, and red colorant. The antenna is matched between 1.5 and 5 GHz (Figure 10a). The measured reflection coefficient is very similar to the simulated one, using the proposed model. The antenna on the gel was positioned in an anechoic planar range facility (Figure 10b), and a vector network analyzer was used to measure the transmission coefficient between the antenna under test and the probe antenna.

At working frequencies, typical near field probes based on open waveguides are bulky and heavy. Because of this, two sets of patch antennas matched at 2, 3 and 4 GHz were manufactured. At these frequencies, the antennas exhibit a gain of 4.1, 5.0 and 5.9 dBi respectively. One set was used as the probe antenna, while the other was used as the reference.

**Figure 11:**
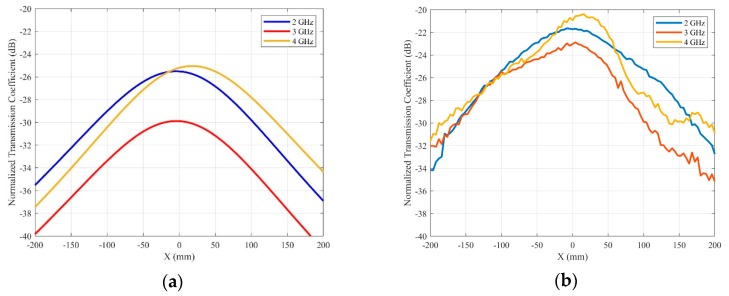
Normalized transmission coefficient for the back radiation of the Jerusalem cross slot antenna. (**a**) Simulations. (**b**) Measurements.

The transmission coefficient along the *X-axis* was simulated and measured for the antenna under test and the reference antennas at a distance of 155 mm. The results were normalized to the transmission coefficient of an isotropic radiator and they are plotted in Figure 11. For all the considered frequencies, the maximum radiation is below −20 dBi, being, in the worst case, 4 GHz, as was predicted in the previous section. Similar results were found in measurement along the *Y-axis*. These results confirm the low level of back radiation of the purposed antenna. 

## 4. Conclusions

In this work, an epidermal wideband antenna for medical radiometry is presented. Body areas without adipose layers, such as the neck or foot, are intended as matching body tissues. A new, compact, low-profile and low-cost antenna, based on two radiating slots over a flexible substrate layer, was designed and validated. The antenna can reduce the backward radiation by using a short-circuited stub. The manufactured prototype was proved with four different volunteers, matched to body areas such as the carotid, the instep, and the sole of the foot. In all cases, the antenna was matched, showing a wideband performance between 1.8 and 5 GHz, that is around a 100% relative bandwidth. In addition, the antenna has very low back radiation up to 4 GHz, which reduces interferences and minimizes its contribution to the radiometric temperature. A measurement campaign in an anechoic facility was carried out, and it was shown that the normalized transmission coefficients are below −20 dBi in all cases. The designed antenna is easy to manufacture and comfortable for patients. It has a simple and flexible design to be used on other areas of the body, and it could be easily integrated into wearable microwave sensors. 

## Figures and Tables

**Table 1:** Design Parameters of the Jerusalem Cross Antenna (in mm).

*W*_1_	*W*_2_	*L*_1_	*L*_2_	*L_s_*	*W*_0_
11.0	6.0	8	4	1.4	1.2

**Table 2:** Dielectric properties of the tissues [18].

Tissue	Frequency (GHz)	ε_r_	σ (S/m)
Wet Skin	2	43.6	1.34
Wet Skin	4	40.8	2.70
Muscle	2	53.3	1.45
Muscle	4	50.8	3.02

**Table 3:** Calculated weighting functions and SAR for the skin, for a power of 15 mW.

Frequency	Back-Rad. *W_back_*	*W*_1_ (0–3 mm)	*W*_2_ (3–7 mm) (VUI)	*W*_3_ (Z > 7 mm)	Skin SAR (W/kg)
2 GHz	0.028	0.571	0.165	0.173	15.4
3 GHz	0.009	0.623	0.195	0.163	16.5
4 GHz	0.036	0.668	0.175	0.071	18.0

**Table 4:** Body Mass Index of the Volunteers.

Volunteer	Gender	BMI	Foot Size (cm)
S1	Female	17.4	25.5
S2	Female	21.8	22.5
S3	Male	30.2	29
S4	Male	18.3	27.5
